# IL-21 Limits Peripheral Lymphocyte Numbers through T Cell Homeostatic Mechanisms

**DOI:** 10.1371/journal.pone.0003118

**Published:** 2008-09-05

**Authors:** Shrimati Datta, Nora E. Sarvetnick

**Affiliations:** Department of Immunology, The Scripps Research Institute, La Jolla, California, United States of America; New York University School of Medicine, United States of America

## Abstract

**Background:**

IL-21, a member of the common γ-chain utilizing family of cytokines, participates in immune and inflammatory processes. In addition, the cytokine has been linked to autoimmunity in humans and rodents.

**Methodology/Principal Findings:**

To investigate the mechanism whereby IL-21 affects the immune system, we investigated its role in T cell homeostasis and autoimmunity in both non-autoimmune C57BL/6 and autoimmune NOD mice. Our data indicate that IL-21R knockout C57BL/6 and NOD mice show increased size of their lymphocyte population and decreased homeostatic proliferation. In addition, our experimental results demonstrate that IL-21 inhibits T cell survival. These data suggest that IL-21 acts to limit the size of the T cell pool. Furthermore, our data suggest IL-21 may contribute to the development of autoimmunity.

**Conclusions/Significance:**

Taken together, our results suggest that IL-21 plays a global role in regulating T cell homeostasis, promoting the continuous adaptation of the T cell lymphoid space.

## Introduction

The IL-21 cytokine has been implicated in the development and maintenance of numerous autoimmune and inflammatory diseases in both animal and human models [1]. IL-21 is produced by CD4+ T cells and natural killer T cells under autocrine regulation and functions through the IL-21 receptor (IL-21R) [2], [3]. The IL-21R, which consists of a private IL-21R-α chain and the shared common γ-chain, is widely expressed on T cells, B cells, NK cells, and dendritic cells in all lymphoid tissues, as well as some non-immune cells, such as fibroblasts and epithelial cells [4].

IL-21 has been shown to act as a pro-inflammatory Th1-promoting cytokine. In addition, it has also been shown to inhibit the differentiation of Th1 cells [5], [6], [7]. Furthermore, several recent reports have shown that IL-21 is expressed at much higher levels in Th17 cells, a distinct Th lineage that is thought to mediate tissue inflammation [3], [8], [9], [10].

Depending on the specific activation signals received, IL-21 can either promote or inhibit lymphoid proliferation [7], [11]. IL-21 has been shown to promote apoptosis among NK, B and T cell populations, although one report demonstrated increased T cell survival following exposure to IL-21 [7], [12], [13], [14], [15]. IL-21 also promotes T cell effector function, measured by increased CTL activity and IFNγ production [9], [13], [16].

Previously, we reported that the expression levels of IL-21 and its receptor were increased in the autoimmune prone NOD mouse strain compared to mice that contain the corresponding protective interval from C57BL/6 mice. In addition, we reported that NOD mice were mildly lymphopenic and contained a higher proportion of mitotically active T cells in their periphery [17]. In the present study, we have investigated the role of the IL-21R in T cell homeostasis using mice on either the non-autoimmune prone C57BL/6 background or on the autoimmune prone NOD background. Importantly, our data demonstrate a requirement for the IL-21R in the regulation of T cell numbers in the periphery and potentially in the perpetuation of pancreatic autoimmunity.

## Results

### Increased size of the peripheral T cell pool in IL-21R-deficient mice

**Figure 1:**
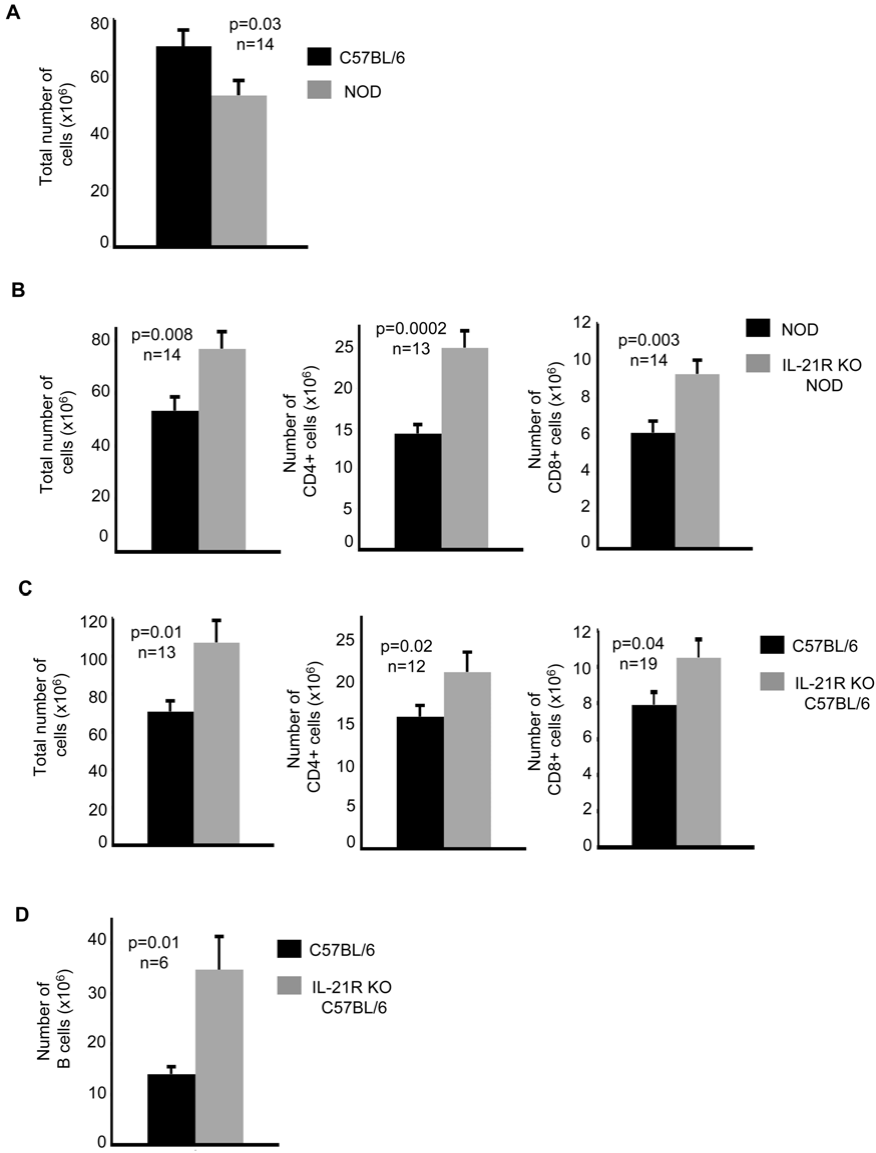
Increased cell numbers in IL-21R-deficient mice. (A) Total splenocyte numbers in 6 to 8-week old C57BL/6 mice compared to age-matched NOD mice (n = 14 mice/group, p = 0.03). (B) Total cell numbers (n = 14 mice/group, p = 0.008), absolute CD4+ T cell numbers (n = 13 mice/group, p = 0.0002) and absolute CD8+ T cell numbers (n = 14 mice/group, p = 0.003) for the splenocytes of NOD mice, at 6 to 8-weeks of age, compared to those from age-matched IL-21R KO NOD mice. (C) Total cell numbers (n = 13 mice/group, p = 0.01), absolute CD4+ T cell numbers (n = 12 mice/group, p = 0.02), and absolute CD8+ T cell numbers (n = 19 mice/group, p = 0.04) from the spleens of C57BL/6 mice, average age of 8 weeks, and IL-21R KO C57BL/6 mice, average age of 9 weeks. (D) Absolute B cell numbers (n = 6 mice/group, p = 0.01) for mice described in C. Data shown are from three or four independent experiments. Results are presented as mean absolute numbers ±SEM.

NOD mice have been shown to be mildly lymphopenic [17], albeit with some controversy [18]. In support, we observed a decrease in the total number of cells in NOD mice compared to C57BL/6 mice (Fig. 1A). Since IL-21 has been shown to promote lymphoid death in some recent studies [1], [14], [19], we asked whether IL-21 expression in NOD mice leads to the reduced T cell numbers observed in these mice. To test this hypothesis we counted the numbers of cells within the lymphocyte subsets of the spleens in normal and IL-21R-deficient NOD mice. Our studies revealed that the total lymphocyte numbers, as well as the numbers of CD4 and CD8 T cells, were significantly increased in IL-21R-deficient NOD mice compared to control NOD mice (Fig. 1B).

To determine whether this is a global effect, we asked if IL-21 affects lymphocyte numbers in the non-autoimmune prone C57BL/6 strain. Our results demonstrate a significant increase in the number of total splenocytes and in T cell subsets, as indicated by increased absolute numbers of CD4 and CD8-positive cells in IL-21R KO C57BL/6 mice compared to age-matched control C57BL/6 mice (Fig. 1C). In addition, we found an increase in the absolute numbers of B220-positive cells in IL-21R KO C57BL/6 mice compared to control mice, confirming published reports that IL-21 induces B cell apoptosis (Fig. 1D) [14]. Our results clearly show that IL-21 exposure acts to limit the numbers of lymphocytes in the periphery in NOD and C57BL/6 mice, suggesting a general role for IL-21 in regulating the size of the T cell pool.

### Reduced proportions of proliferating T cells in IL-21R-deficient mice

**Figure 2:**
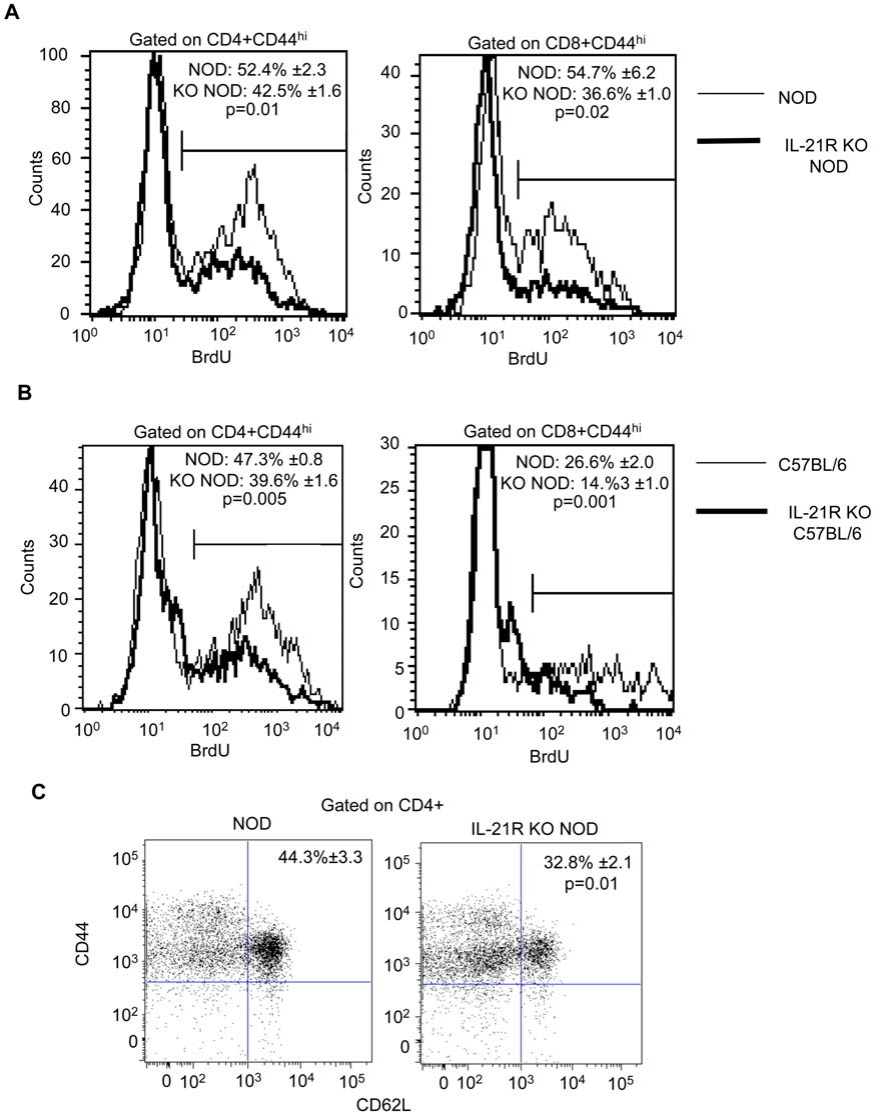
Reduced T cell proliferation in IL-21R-deficient mice. Representative histogram plots for individual NOD (thin line) and IL-21R KO NOD (thick line) mice showing flow cytometric analysis of BrdU incorporation in CD4+CD44^hi^ and CD8+CD44^hi^ populations after 5 days of BrdU treatment. The data, obtained from analyzing the spleens of 7 to 9-week-old NOD and age-matched IL-21R KO NOD mice, is presented as mean percentages of BrdU+ cells ±SEM from one experiment with a total of n = 4 mice/group. The experiment, using the same number and age of mice, was repeated twice with similar results. (B) Representative flow cytometric analyses of splenocytes from individual C57BL/6 (thin line) and IL-21R KO C57BL/6 (thick line) mice showing BrdU incorporation in CD4+CD44^hi^ and CD8+CD44^hi^ T cells after 5 days of BrdU treatment. Mean percentages of BrdU+ cells ±SEM are shown from one experiment with a total of n = 4 mice/group with an average age of 10–12 weeks. The experiment was repeated three times with similar results. For both A and B, the gate on the histogram plots represents isotype control staining. (C) Representative dot plot showing expression of CD44 and CD62L in the CD4+ population of individual NOD and IL-21R KO NOD mice. Pooled data ±SEM from a total of n = 8 mice/group, at 7 to 9-weeks of age, from two independent experiments is shown. Gates are based on isotype control staining.

The level of T cell proliferation is regulated in part by the size of lymphoid compartments, reflecting the innate drive to maintain a full pool of peripheral lymphocytes [20], [21]. We hypothesized that the overall increase in peripheral T cell numbers in IL-21R-deficient mice could inhibit T cell proliferation in the periphery. To test this hypothesis, BrdU was administered in the drinking water of IL-21R-deficient and control NOD and C57BL/6 mice. The proportion and subset identities of the proliferating T cells within the spleen were determined by FACS. Since actively dividing cells upregulate CD44 [22], we gated on the CD44^hi^ population of the CD4 and CD8 T cell subsets. We found that the proportion of these T cells incorporating BrdU in the periphery was significantly reduced in the absence of the IL-21R in both the NOD and C57BL/6 strains (Fig. 2A and B). Homeostatically dividing cells also express high levels of CD62L, whereas the levels of this antigen are reduced in conventionally activated T cells [17]. Thus, CD44^hi^CD62L^hi^ cells represent homeostatically proliferating cells and we found a decrease in this T cell population in the IL-21R deficient NOD mice compared to NOD mice (Fig. 2C). These results indicate that the proportion of mitotically active cells that are homeostatically dividing is reduced in IL-21R-deficient NOD and C57BL/6 mice.

### IL-21R KO T cells expand normally upon encounter with self-antigen

**Figure 3:**
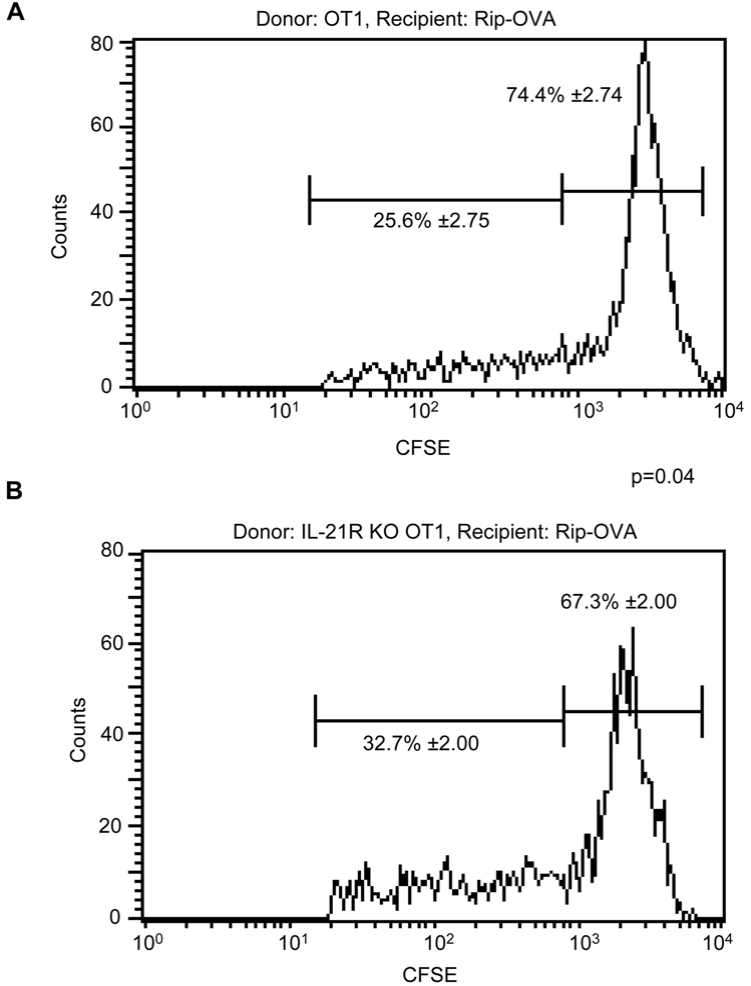
Expansion of IL-21R-deficient T cells upon encounter with self-antigens. CFSE-labeled splenocytes from 6 to 8-week-old (A) OT1 or (B) IL-21R KO OT 1 mice were adoptively transferred into wild-type Rip-OVA recipient mice, with an average age of 8-weeks, for five days. Cells were then recovered from the pancreatic lymph nodes of recipient mice and analyzed by flow cytometry for CFSE dilution to measure proliferation. A representative CFSE dilution profile of the indicated Vβ5.1+/5.2+ (donor) T cells from individual recipient mouse is shown. The data shown reflects the mean percentage of CFSE^hi^ and CFSE^lo^ T cells ± SEM for a total of n = 15 mice/group from four independent experiments, p = 0.04. Analysis of cells before transfer indicated >80% were naïve (CD8+CD44l^o^, data not shown).

Our results indicate that lack of the IL-21R leads to an increase in lymphocyte numbers and a decrease in homeostatic T cell proliferation in the periphery of NOD and C57BL/6 mice. The reduced proliferation may be explained by an intrinsic defect in the proliferation of IL-21R-deficient T cells, or by the lack of space and growth factors in the IL-21R-deficient periphery. To test whether IL-21R-deficient T cells have a defect in their proliferative capacity, we backcrossed the IL-21R-deficient C57BL/6 mouse with the OT1 TCR transgenic strain, which contains a MHC class1-restricted T cell receptor specific for an ovalbumin peptide, to derive homozygous IL-21R KO OT1 transgenic mice. Since the ovalbumin antigen is absent from normal mice, OT1 transgenic mice harbor a monoclonal population of phenotypically naïve TCR transgenic T cells [23]. We adoptively transferred CFSE-labeled T cells from both IL-21R KO OT1 and control OT1 mice into recipient Rip-OVA mice, a strain that expresses ovalbumin in the pancreatic islets. Importantly, this protocol eliminates critical experimental caveats, since the genetically identical recipient mice have equivalent numbers of peripheral lymphocytes. We found that the CFSE-labeled donor IL-21R KO OT1 T cells from the pancreatic lymph node (PLN) were able to proliferate significantly more than the control CFSE-labeled donor OT1 T cells within Rip-OVA recipients (Fig. 3A and B). This result demonstrates that IL-21R KO cells do not have an intrinsic defect in proliferation when exposed to antigen. Similarly, we also observed that both of these populations proliferated in Rag1-deficient recipients (data not shown), which do not have any endogenous lymphocytes and provide an empty environment conducive to homeostatic expansion [24]. The IL-21R KO cells showed a modest advantage over wild-type controls (data not shown). Thus, IL-21R deficient T cells show no defect in T cell expansion, which indicates that the decreased T cell proliferation observed in Figure 2 arises from the lymphoid environment of IL-21R KO mice.

### Increased T cell survival factors in IL-21R-deficient mice

**Figure 4:**
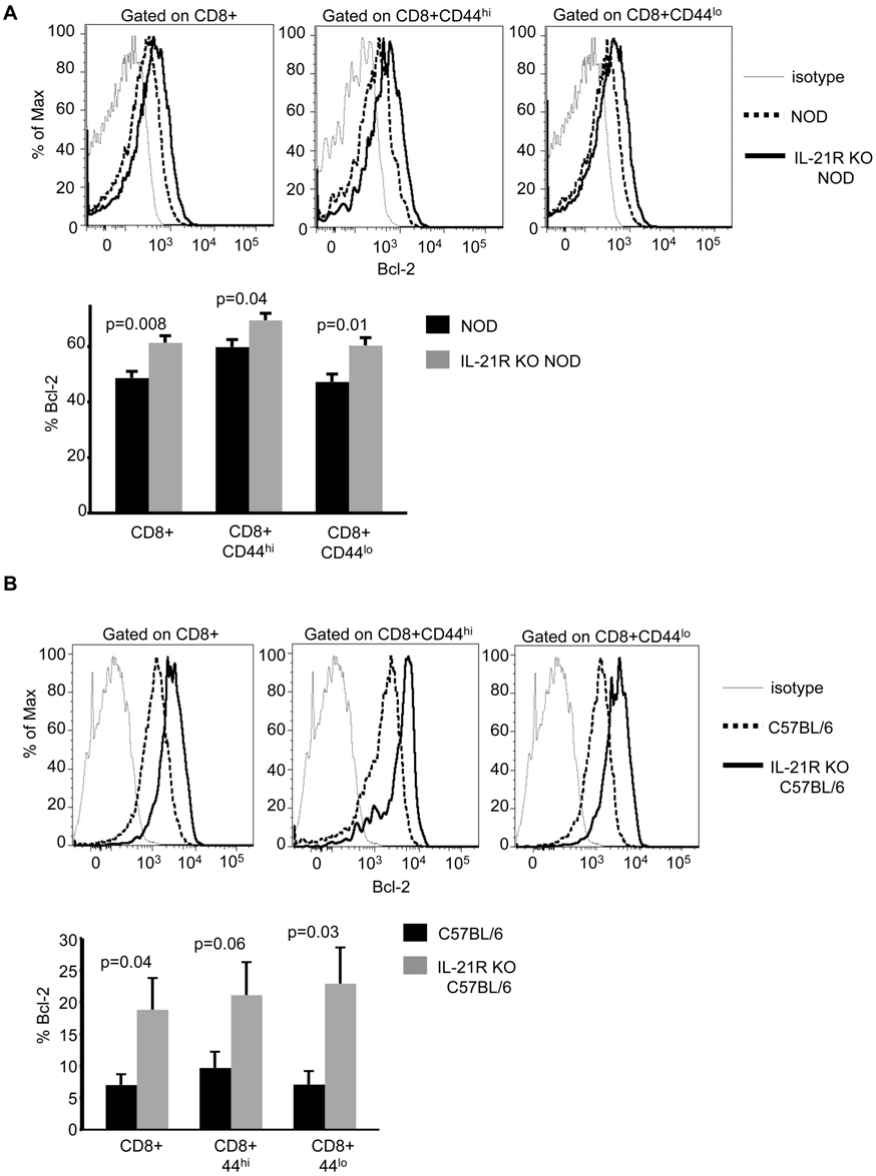
Increased T cell survival factors in IL-21R-deficient mice. (A) Representative histograms showing total Bcl-2 expression in indicated CD8 populations for individual NOD (dashed line) and IL-21R KO NOD (solid line) mice relative to isotype control staining (gray line). Below, the graph represents Bcl-2 flow cytometric data ±SEM for one of two independent experiments using n = 4 mice/group. Similar results were found in both experiments. Pancreatic lymph nodes of NOD mice with an average age of 13-weeks and age-matched IL-21R KO NOD mice were analyzed. (B) Levels of Bcl-2 expression in the indicated splenocyte subsets for individual C57BL/6 (dashed line) and IL-21R KO C57BL/6 (solid line) mice are shown in representative histograms relative to isotype control staining (gray line). Below, graphical representations of the percentage of Bcl-2 expression ±SEM pooled from three independent experiments with a total of n = 12 mice/group. 6-8-week-old C57BL/6 mice and aged-matched IL-21R KO C57BL/6 mice were used.

Some recent studies have demonstrated that unlike the pro-survival functions of other common γ-chain-associated cytokines, such as IL-7, IL-21 exposure can cause cell death [14], [19], [25]. We therefore hypothesized that the increased numbers of lymphocytes in IL-21R-deficient NOD and C57BL/6 mice might result from increased T cell survival. In support of this hypothesis, we found increased levels of the pro-survival protein Bcl-2 in total, activated (CD44^hi^) and resting (CD44^lo^) CD8 T cells in the PLN of IL-21R-deficient NOD mice and in splenocytes of IL-21R KO C57BL/6 mice, compared to controls for both strains (Fig. 4A and B). Although the increased Bcl-2 expression in the CD44^hi^ population of IL-21R KO C57BL/6 mice did not reach the 0.05 level of significance, the trend is consistent with the other cell populations.

**Figure 5:**
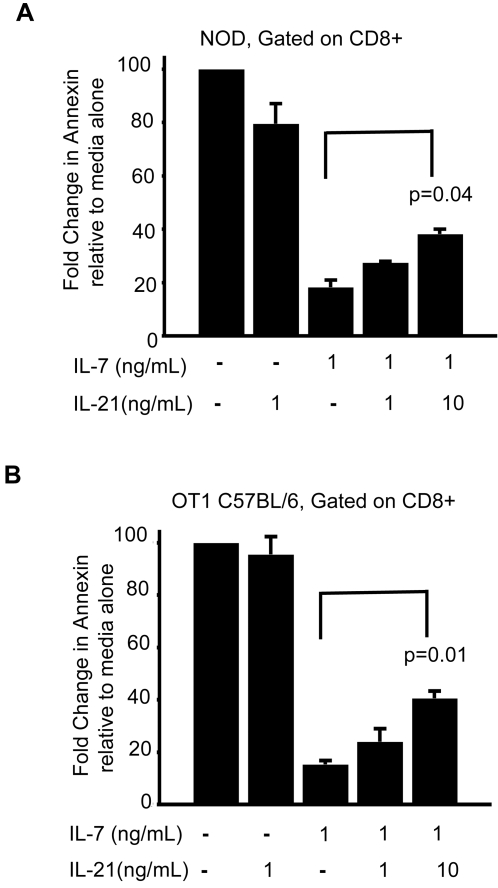
IL-21 antagonizes the pro-survival effects of IL-7 *in vitro*. Analysis of the effects of IL-21 on T cell survival in the presence or absence of IL-7. Cells from the spleen and pancreatic lymph nodes of NOD or OT1 C57BL/6 mice were cultured with IL-7 (1 ng/ml) and/or IL-21 (1 ng/ml or 10 ng/ml) as described in Materials and Methods. Graphical representation of Annexin V staining by FACS analysis for CD8+ T cell populations from (A) NOD and (B) OT1 C57BL/6 mice. Percentage of Annexin V+ cells cultured in media alone (no cytokine treatment) was set at 100 and Annexin V+ cells treated with cytokine are represented as fold change relative to media alone ±SEM. Percentage of Annexin V+ cells cultured with IL-21 by itself at 10 ng/ml is similar to cells cultured with IL-21 at 1 ng/ml (data not shown). The data shown is pooled from two independent experiments.

The cytokine milieu *in vivo* is complex, with competition between the different γ- chain dependent cytokines determining survival or death. To determine how IL-21 affects cell survival in the presence of pro-survival cytokines we performed *in vitro* studies in the presence and absence of the critical pro-survival cytokine IL-7 [26]. Confirming previous reports, exposure of NOD and OT1 C57BL/6 CD8 T cells to IL-7 resulted in decreased Annexin and increased Bcl-2 expression compared to cells cultured without this cytokine [27]. In contrast, treatment with IL-21 failed to inhibit the expression of Annexin or induce Bcl-2 expression in comparison to cells cultured in the absence of cytokine (Fig. 5A and B, and data not shown). Importantly, IL-21 exposure partially blocked the pro-survival activity of IL-7 in NOD and OT1 C57BL/6 CD8 T cells in a concentration dependent manner (Fig. 5A and B). Our results indicate a similar trend with CD4 NOD T cells treated with these cytokines *in vitro* (data not shown). Taken together, our data suggest that IL-21 inhibits the pro-survival function of IL-7, thereby limiting the size of the lymphoid compartment.

### Reduced effector function in IL-21R-deficient NOD mice

**Figure 6:**
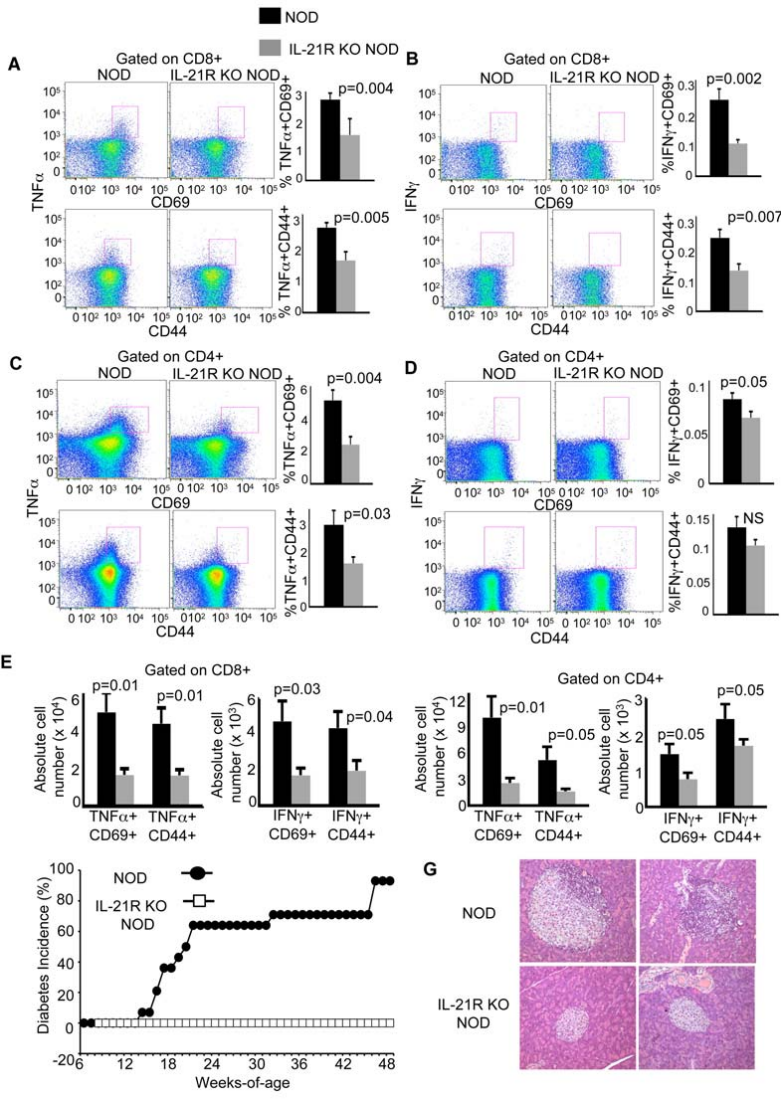
Decreased T cell effector function in IL-21R-deficient NOD mice. Representative dot plots from individual NOD and IL-21R KO NOD mice showing frequency of (A) TNFα+/CD69+, TNFα+ /CD44+, (B) IFNγ+ /CD69+, IFNγ+/CD44+ populations in CD8+ T cells and (C) TNFα+/CD69+, TNFα+/CD44+, (D) IFNγ+/CD69+, IFNγ+/CD44+ populations in CD4+ T cells. Gates are based on isotype control staining. Graphs represent pooled flow cytometric data from pancreatic lymph nodes of 11-to-13 week old mice for two independent experiments with a total of n = 8 mice/group. Results are presented as the mean percentages ±SEM. (E) Percentages of the indicated cell populations from A–D were multiplied by the total number of cells in the pancreatic lymph nodes for each individual animal to give the absolute numbers for each indicated cell population. Data are shown as mean absolute numbers ±SEM. (F) Cumulative incidence of diabetes was monitored by measuring blood glucose levels in NOD (close circles) and IL-21R KO NOD (open squares) mice (n = 15 mice/group) at the indicated ages. (G) Paraffin sections of pancreata from NOD and IL-21R KO NOD mice stained with H&E. In total, four NOD and four IL-21R KO NOD mice were evaluated, with fifty individual islets examined per strain. The images shown in these panels were obtained from 2 different mice per strain; the NOD mice were 11 weeks of age; the IL-21R KO NOD mice were 13 weeks of age.

IL-21 can promote effector function and Th1 type cytokine secretion [6], [16]. Therefore, we asked whether T cell effector function was diminished compared to controls by measuring the frequency of TNF-α or IFN-γ-expressing T cells in activated cells isolated from IL-21R KO and age-matched NOD mice. We found a reduction in the frequency of CD69- and CD44-expressing CD8 or CD4 T cells spontaneously producing TNFα or IFNγ in the PLN of IL-21R KO NOD mice compared to control NOD animals (Fig. 6A–D). In addition, we observed reduced absolute numbers of these cell populations expressing TNF-α or IFN-γ in IL-21R KO NOD mice compared to control NOD animals (Fig. 6E). Our results demonstrate that the proportion and number of effector T cells is reduced in IL-21R KO NOD mice compared to age-matched NOD mice.

The acquisition of T cell effector function promotes islet destruction during the development of diabetes [28]. Since T cell effector function was diminished when the IL-21R was absent, we asked whether IL-21 plays a role in precipitating clinical diabetes in NOD mice. We tested whether NOD mice lacking the IL-21R are protected from disease by measuring blood glucose levels in control and IL-21R KO NOD mice. We found that NOD mice deficient in the IL-21R were completely protected from diabetes (n = 15, 0% incidence) during the 48-week observation period (Fig. 6F). Furthermore, pancreatic islet infiltration precedes clinical diabetes, and is a hallmark of the autoimmune process. To test whether infiltration of the pancreatic islets was inhibited in the IL-21R KO NOD mice, we performed a comparative histological analysis on the pancreata from the two groups of mice. We found that the IL-21R-deficient NOD islets had no detectable inflammation (Fig. 6G). The absence of diabetes and insulitis suggests that islet autoimmunity is decreased when IL-21 responses are absent.

## Discussion

In this study, we demonstrate that IL-21 has a major impact on the immune systems of both autoimmune prone and normal mice. Our experimental results demonstrate that IL-21 negatively regulates lymphocyte numbers in the periphery, perhaps by tempering the pro-survival actions of IL-7. Our data demonstrate that IL-21 plays a central role in lymphocyte homeostasis, balancing survival and turnover to foster the continuous evolution of the T cell lymphoid space.

Lymphocyte numbers are controlled by both pro-survival and proliferative activities of several key growth factors [29], [30]. A previous study reported no defect in lymphocyte numbers in IL-21R-deficient mice, which may be explained by the difference in mouse strains used compared to this study [31]. To analyze the pro-survival activities of cytokines, we measured the expression of the apoptotic marker Annexin V, and the pro-survival marker Bcl-2. We addressed both the *in vivo* role of IL-21 in T cell survival and also the function of IL-21 in T cell survival in relation to other cytokines using an *in vitro* assay. We observe that IL-21 can reverse the pro-survival functions of IL-7 *in vitro*, as documented by comparing both Annexin V and Bcl-2 expression. Annexin, a phospholipid-binding protein that binds to membrane phospholipid phosphatidylserine, is released into the external cellular environment in apoptotic cells, whereas Bcl-2 expression is regulated by cytokine signaling through the JAK-STAT pathway, blocking apoptotic signals [32], [33]. Taken together, analysis of two separate markers provides strong evidence that IL-21 opposes the known pro-survival cytokine IL-7 [34], [26].

Since both IL-21 and IL-7 signal through the common γ-chain, it is possible that these factors compete for limiting amounts of this receptor chain within lymphocytes. In addition, IL-21 has been shown to activate the JAK-STAT intracellular signaling pathway distinctly from other common γ-chain cytokines, including IL-7 and IL-15, which activate STAT5 [6], [35]. Since activation of STAT5 promotes survival pathways, including the induction of Bcl-2, the absence of STAT5 activation by IL-21 could be responsible for its lack of pro-survival effects [33]. Thus, IL-21 may block IL-7 action by preventing the phosphorylation of STAT5, thereby blocking the induction of Bcl-2. In support of this idea, B cell death by IL-21 was prevented by over-expression of Bcl-2 in C57BL/6 mice [12]. Interestingly, STAT1, through which IL-21 also signals, has been shown to reduce the expression of Bcl-2 [38].

In addition, IL-21, unlike IL-7, has been shown to activate Stat3 [35]. Stat3 has been associated with apoptosis, where phosphorylated Stat3 activates the p50a and p55a regulatory subunits of PI3 kinase, which reduce the levels of phosphorylated Akt/PKB kinase and prevent the pro-survival effect of Akt/PKB [36]. In contrast, IL-7 has been shown to induce the activity of Akt/PKB, which leads to the phosphorylation and sequestration of the pro-apoptotic protein BAD. This in turn prevents BAD from interacting with and inhibiting Bcl-2, thereby allowing the pro-survival activity of Bcl-2 [37]. Thus, it is possible that IL-21 suppresses the pro-survival effects of IL-7 by inducing STAT3 phosphorylation, which would ultimately prevent the downstream signaling events necessary for IL-7 mediated survival. IL-21 has also been shown to activate other pro-apoptotic molecules, such as BIM and BID [19]. Our observations are consistent with other reports indicating that IL-21 can induce apoptosis in lymphocytes [14], [19], [27], [39]. IL-21-mediated induction of T cell survival reported in a previous study may be due to the concentration of IL-21 *in vitro*, where cell viability was enhanced at higher concentrations of IL-21 [15].

Since cytokines are central in regulating T cell homeostasis, the actions mediated by IL-7 and IL-21 are physiologically relevant. The process of T cell homeostasis acts to maintain a constant level of lymphocytes in the immune system through the regulation of lymphocyte survival and death. Improper regulation of T cell homeostatic mechanisms, such as IL-21 inhibition of T cell survival, can result in decreased numbers of lymphocytes, or lymphopenia, as observed in both NOD and C57BL/6 mice compared to IL-21R KO animals of both strains. In NOD mice, impaired T cell homeostatic mechanisms along with the presence of islet-reactive T cells contribute to disease progression, as discussed in greater detail below.

In addition to survival mechanisms, T cell numbers in the periphery are also regulated by the extent of proliferation that can be affected by both intrinsic and environmental factors *in vivo*. All of the common γ-chain binding cytokines can induce aggressive mitosis of T cells *in vitro* [29], [40]. Supporting this idea, IL-21 induced *in vitro* proliferation in the presence of cytokines such as IL-7 and IL-15, and could induce modest amounts of proliferation on its own [16], [39], [40]. Conversely however, another study demonstrated that IL-21 blocks IL-15 dependent expansion of activated CD8+ T cells *in vitro* [13]. It is important to note that *in vitro*, T cell proliferation is measured in the absence of the microenvironmental pressures induced by lymphoid space.

*In vivo* the steady state proliferation of lymphocytes is dependent on the size of the peripheral lymphoid pool. Under conditions of low T cell numbers, the increased availability of growth factors induces proliferation to increase the size of the lymphoid pool [29]. Since we observed a significant increase in the number of lymphocytes in the absence of the IL-21R, the most straightforward explanation for our results is that proliferation is inhibited due to the increased size of the T cell pool in the periphery, a situation that would limit the amount of mitosis. Thus, some of the discrepancy that has arisen regarding the mitotic actions of IL-21 may be a result of the distinct *in vivo* and *in vitro* approaches utilized, where homeostatic environmental factors are differentially available.

Although we observed decreased proliferation in IL-21R KO NOD and C57BL/6 mice, our results do not support a mechanism whereby IL-21R-deficient T cells are simply inhibited in their ability to proliferate. Using the OT1 adoptive transfer system we found that following transfer into normal and immunodeficient hosts, IL-21R KO T cells are not inhibited in their ability to proliferate, and are fully capable of both antigen-stimulated and homeostatically-induced proliferation. Our CFSE dilution results further suggest that the IL-21R-deficient cells may even have enhanced proliferative capacity *in vivo*. This may be mediated by a reduced intercellular competition for the common γ-chain, and enhanced ligation of IL-2, IL-7 and IL-15, prolonging the proliferative response. This idea is consistent with the observation that blocking IL-21 does not inhibit the proliferative response that occurs upon IL-21 addition, suggesting that IL-21 may act indirectly to promote proliferation, perhaps through discouraging ligation of growth promoting cytokines [41]. Our data also support a previous study demonstrating that IL-21R-/- mice do not show defects in T cell proliferation [8].

Our data also indicate that when the IL-21R is absent, the proportion and numbers of effector T cells is diminished. This result supports previous work where IL-21 has been shown to induce pro-inflammatory cytokine secretion during rheumatoid arthritis and increase IFN-γ production in a murine model for experimental autoimmune encephalomyelitis (EAE) [7], [42]. The pro-inflammatory effects of IL-21 may directly arise through its known activation of the STAT3 pathway, which stimulates the production of pro-inflammatory cytokines [4], [8]. Recent findings that Th17 cells produce IL-21 further support the connection between IL-21 and inflammation [3], [8], [10].

Our previous studies of NOD mice led us to hypothesize that elevated IL-21 levels contribute to the development of disease by creating a lymphopenic environment that drives homeostatic expansion and autoimmunity [17]. In this study, we found that IL-21 promotes insulitis and diabetes in NOD mice. It is well established that diabetogenic T cells express high levels of effector cytokines, such as IFN-γ and TNF-α [43], [44], [45]. The decreased number of effector T cells we observed in IL-21R deficient mice underlies the protection against diabetes in the absence of IL-21 signaling. Furthermore, homeostatically expanding cells in NOD mice have also been shown to rapidly acquire effector function [17]. In addition to T-cell mediated pathogenesis of type 1 diabetes, B cells have also been shown to contribute to disease pathogenesis in the form of auto-antibody production or presentation of self-antigen to T cells thus promoting T-cell mediated disease [46]. Auto-antibodies to antigens such as glutamate decarboxylase (GAD) and insulin have been reported in both NOD mice and type 1 diabetic patients although the direct pathogenic role of auto-antibodies is still controversial [47]. Recent studies have shown that IL-21 regulates the generation of T follicular help cells (Tfh) which in turn impact B cell function, such as in germinal center reactions [48], [49]. Germinal center reactions are present in autoimmune diabetic mice and likely to be the site where self-reactive auto-antibodies are generated [50]. Tfh function on B cells could contribute to the protection from disease we observe in IL-21R KO mice, where decreased Tfh function in the absence of IL-21R could lead to decreased B cell responses. Furthermore, it should be noted that it is possible that diabetes resistance in IL-21R-deficient NOD mice may be attributed to still unidentified protective alleles in the C57BL/6 genome, as has been identified in disease-resistant IFNγ knockout NOD mice [51]. Despite this caveat, our studies in NOD and C57BL/6 mice clearly indicate that IL-21 reduces T cell survival and suggest that it might promote lymphopenia to drive homeostatic proliferation. Homeostatically expanding cells in NOD mice probably comprise islet-reactive T cells with acquired effector function, whereas in C57BL/6 animals, IL-21 signaling appears to represent a normal mechanism involved in steady-state maintenance of peripheral T cell numbers.

Decreased T cell longevity has been causally associated with the development of autoimmunity. It has been shown that over-expression of Bcl-2 in both T and B cells leads to protection from insulitis in NOD mice [52]. There is also clinical support for this concept, since insulin-dependent diabetes mellitus (IDDM) patients show significantly reduced Bcl-2 expression in CD3+ and memory CD4+CD45RO+ populations and have higher levels of spontaneous apoptosis [53]. Furthermore, patients with systemic lupus erythematosus (SLE) have increased numbers of apoptotic lymphocytes and macrophages and exhibit decreased cell survival compared to control subjects [54].

IL-21 has been associated with the development and maintenance of numerous autoimmune and inflammatory diseases in both animal and human models. For example, polymorphisms of IL-21 and its receptor were identified in patient samples associated with Type 1 diabetes (53). Furthermore, increased IL-21R levels have been reported in patients with rheumatoid arthritis, systemic sclerosis, and inflammatory bowel disease, including Crohn's disease and ulcerative colitis (39, 54–56). Moreover, the BXSB-Yaa mouse model of SLE expresses elevated circulating levels of IL-21 (23). Other interesting reports have shown that *in vivo* neutralization of IL-21 can lead to decreased rheumatoid arthritis in rats and lupus development in mice, as well inhibition of matrix metalloproteinase secretion in inflammatory bowel disease (18, 52, 57). Interestingly, IL-21 activity is increased in Helicobacter pylori infection, leading to higher production of matrix metalloproteinases MMP2 and MMP9, and MMP2 and MMP9 have been shown to be upregulated in Type 1 diabetes (58, 59).

There have been various explanations for autoimmunity resulting from the improper elimination of T cells with a high affinity for self-antigen. For example, it has been proposed that autoreactive T cells are part of the normal repertoire, but are maintained and do not elicit their autoreactive capabilities due to the presence of T regulatory cells [55]. Indeed, the introduction of T regulatory cells, characterized as CD4+CD25+, has been shown to alleviate autoimmune disease in various experimental models, such as EAE [56]. Interestingly, CD4 T cells are resistant to suppression by CD4+CD25+ T regulatory cells in the presence of IL-21 [57]. In addition, IL-21 is able to suppress T regulatory cells and promote the induction of Th17 cells [8].

Our data provides another aspect critical in the regulation of autoimmunity by IL-21, which can negatively impact the size of the lymphoid compartment. Dysregulation of T cell homeostasis can result in lymphoproliferative disorders or lymphopeina, both of which are associated with pathological states (40). Indeed, in addition to Type 1 diabetes, lymphopenia is characteristic of several human diseases that also exhibit increased levels of IL-21, such as rheumatoid arthritis, Crohn's disease, SLE, and Sjogren's syndrome [58]. To compensate for the lack of T cells, proliferation occurs that favors expansion and activation of auto-reactive T cells that can lead to autoimmunity [21]. In rheumatoid arthritis, decreased thymic output leads to increased homeostatic proliferation that favors T cells of an auto-reactive nature [59]. Conversely, immune activation via homeostatic proliferation is important for the rejection of tumors and IL-21 administration may inhibit the progression of metastatic melanoma and renal carcinoma in clinical protocols [60], [61]. Therefore, a complete understanding of immune homeostatic mechanisms is important for therapeutic intervention in major diseases affecting humans worldwide. However, the manifestation of autoimmune disease is not solely dependent on lymphoid pool size, since other conditions leading to lymphopenia, such as bone marrow transplantation or chemotherapy, do not always manifest into autoimmunity [55], [58]. Indeed, Krupica et al propose that autoimmunity represents a two-hit model, where lymphopenia together with other insults, such as T regulatory cell depletion, promote autoimmunity [62].

Our results support an association between IL-21 expression and the regulation of T cell numbers in the periphery. Importantly, IL-21 blockade could represent an important therapeutic target to control T cell survival and homeostatic expansion, leading to novel treatment strategies for autoimmune diseases as well as immunodeficiencies.

## Materials and Methods

### Mice

All mice were housed at The Scripps Research Institute Animal Facility (La Jolla, CA). All live animal experiments were approved by the Institutional Animal Care and Use Committee (IACUC) and the Animal Research Committee (ARC) and were conducted in accordance with institutional guidelines for animal care and use. The IL-21R KO C57BL/6 mice were obtained from Dr. Warren Leonard (NIH, Bethesda, MD). IL-21R KO C57BL/6 mice were backcrossed onto the OT1 C57BL/6 transgenic strain to obtain IL-21R KO OT1 C57BL/6 mice. The presence of the OT1 gene was confirmed by PCR. We utilized speed congenic backcrossing and screening techniques to introgress the IL-21R-deficient region from the C57BL/6 background onto the NOD genetic background. After 7 generations, these mice were intercrossed to derive homozygous mutants on the NOD genetic background. We screened 64 polymorphic markers across the genome (autosomes only, X and Y chromosomes were selected by breeding), that included markers for all known regions linked to diabetes susceptibility Idd loci in the NOD mice, plus an additional 4 markers on Chr. 7 flanking the IL-21R mutation to define the size of the congenic interval. N7 mice were NOD-derived at all markers tested across the genome, except for a congenic interval of less than 5 cM flanking the IL-21R-targeted mutation on Chr. 7.

### Immunostaining for Flow Cytometric Analyses

Spleens or pancreatic lymph nodes (PLN) were isolated, processed and stained for FACS analysis as described previously [63]. Antibodies were obtained from eBiosciences and BD Pharmingen. Intracellular staining of Bcl-2, IFNγ, and TNFα (BD Pharmingen) were performed using standard procedures [17]. For intracellular IFNγ and TNFα staining, processed cells from the PLN were stimulated in 96-flat well plates with plate-bound anti-CD3 (20 mg/ml) and anti-CD28 (10 mg/ml), followed by treatment with Golgi plug for 5 hours at 37° prior to flow cytometric staining.

### *In vitro* Cytokine Stimulation

Spleens and PLN from control OT1 or NOD mice were isolated and processed in RPMI media (Hyclone) supplemented with penicillin/streptomycin, glutamine (Gibco), Hepes buffer (Fisher Scientific) and β-mercaptoethanol. 2×10^6^ cells were plated in 48-well plates and cultured for 72 hours at 37° in supplemented RPMI media. Mouse recombinant interleukin-7 (IL-7), at 1 ng/ml (eBioscience), and mouse recombinant interleukin-21 (IL-21), at 1 ng/ml or 10 ng/ml (R & D Systems), were added alone or in combination at the time of culture to the OT1 or NOD cells, after which the samples were incubated for 72 hours at 37°. After culture, cells were harvested, stained for Bcl-2 and Annexin V (BD Pharmingen), and analyzed by FACS as described above.

### BrdU Labeling

Groups of mice were fed BrdU (Sigma) at 0.8 mg/mL in their drinking water for 5 days, with fresh BrdU-containing water provided daily. Following this, the mice were sacrificed, after which the splenocytes were isolated and processed as described previously [17]. After staining with BrdU-specific antibodies and T cell subset markers following the manufacturer's protocol (BD Pharmingen), the T cell subsets incorporating BrdU were identified and quantitated by FACS.

### CFSE Labeling and Adoptive Transfer of T cells

Splenocytes from OT1 or age-matched IL-21R KO OT1 cells were isolated, processed and labeled with CFSE as described previously [63]. 0.5–1×10^7^ CFSE-labeled cells in 200 µL PBS were intravenously injected into Rip-OVA recipient mice. After 5 days, the mice were sacrificed and analyzed for CFSE dilution and cell surface marker staining by FACS. Similarly, CFSE-labeled OT1 cells were injected into Rag1-deficient mice and analyzed by FACS 3 days after adoptive transfer.

### Diabetes and Immunohistochemistry

Blood glucose levels in NOD and IL-21R KO NOD mice were measured weekly using Ascensia ELITE blood glucose test strips (Bayer) and the Ascensia ELITE XL blood glucose meter. Mice with blood glucose readings above 250 mg/dL for two consecutive weeks were classified as diabetic. For histological assessment of islet infiltration, pancreata were fixed in 10% neutral buffered formalin and embedded in paraffin. Sections (4 µm) from five levels through the tissue, separated by at least 120 µm, were stained with H&E.

### Statistical analyses

The Student's t test (unpaired, two-tailed) was used to determine the level of significance of the data. A p value of <0.05 was considered significant. The values corresponding to individual animals from several independent experiments were combined and statistically analyzed to determine p values and the standard error of the mean (SEM).
